# Microbiological and clinical profile of infective endocarditis patients: an observational study experience from tertiary care center Karachi Pakistan

**DOI:** 10.1186/s13019-018-0781-y

**Published:** 2018-09-15

**Authors:** Uzma Shahid, Hasanat Sharif, Joveria Farooqi, Bushra Jamil, Erum Khan

**Affiliations:** 1 Section of Microbiology, Department of Pathology and Laboratory Medicine, Aga Khan University, Karachi, Pakistan; 2 Section of Cardiothoracic Surgery, Department of Surgery, Aga Khan University, Karachi, Pakistan; 3 Section of Infectious Diseases, Department of Medicine, Aga Khan University, Karachi, Pakistan

**Keywords:** Infective endocarditis, Surgical intervention, Microbiological profile, Clinical features, Pakistan

## Abstract

**Background:**

The study analyzed microbiological and antimicrobial susceptibility profile of organisms isolated from patients with infective endocarditis (2015–17) and compared disease outcomes in cohorts of endocarditis patient with history of prior invasive vascular intervention (high risk group) vs those without (native valve group). We hypothesized that high risk group would be more likely to have severe disease outcomes.

**Methods:**

This was a prospective cohort study (2015–17). All blood and cardiac tissue samples of enrolled patients suspected of endocarditis according to modified Duke’s criteria were followed for microbiological and antimicrobial susceptibility profile. The high risk group was compared with the native valve group with 90 day follow up to determine difference in clinical course and outcome in terms of disease severity (defined as any patient with endocarditis undergoing surgical management, readmission or dying). The data was analyzed using SPSS 21.0 software and chi-square test. 90 day mortality was calculated using Kaplan Meier survival curves.

**Results:**

Total 104 patients with endocarditis were enrolled. Overall culture positivity rate was 71.2%. *Streptococcus* species were the most common isolate (36.7%), followed by *S. aureus* (17.3%) cases. In *Streptococcus* species*,* 14.2% showed intermediate susceptibility to penicillin. Thirty six patients were included in the cohort analysis. A poor outcome was seen in 85.7% high risk group as compared to 50% of native valve group. The overall mortality rate was 19.4%.

**Conclusions:**

We found *Streptococcus* species to be the predominant pathogen for endocarditis overall. However *Staphylococcus aureus* predominated native valve group. High risk group showed more complicated clinical course.

**Electronic supplementary material:**

The online version of this article (10.1186/s13019-018-0781-y) contains supplementary material, which is available to authorized users.

## Background

Infective Endocarditis (IE) is associated with significant disease burden, globally. In 2010, IE was associated with 1.58 million disability-adjusted life-years or years of healthy life lost as a result of death and nonfatal illness or impairment [1]. In past few decades the epidemiology, microbiologic profile and treatment outcomes of patients with IE in developed countries have changed significantly. Rheumatic heart disease (RHD) which was once considered the main risk factor for IE is now being superseded by other factors such as invasive vascular interventions (IVI), prosthetic cardiac devices, implants and correction procedures for congenital cardiac defects [1]. Up to 30% of all IE cases have been associated with such factors in developed countries [2]. In Pakistan, there is a mix of population of various socioeconomic strata. While high infant mortality and burden of common infectious diseases including RHD is common in lower strata, there is an expanding population on the other end of the spectrum that is able to afford recent advances in medical care. They have risk factors of IE similar to modern world, such as cardiac implants, invasive vascular interventions and correction of congenital defects. Thus, it is important to study the changing epidemiology and microbiologic profile of patients with endocarditis that may provide better treatment / management strategies in local scenario.

IE studies conducted in Pakistan mainly retrospectively designed; have highlighted younger population being affected more frequently than those reported in the western world [3–6]. As regards etiologic agents, most published studies have limitations in terms of poor sensitivity of bacterial culture methods utilized, such as conventional in-house blood culture methods.; as a result up to 50% of IE cases are reported as culture negative [7, 8]. Lack of authentic data of the common etiologic agents and their susceptibility pattern seriously hampers the choice of empirical antibiotic treatment. Moreover, there is a complete dearth of information that correlates use of vascular / cardiac procedure and implants with IE, disease severity and treatment outcomes in Pakistan.

IE patients with cardiac interventions / prosthetic valves have worse outcomes or complicated disease course compared to patients with native valves. A study in Cleveland compared long term post-surgery survival amongst patient with endocarditis having native valve endocarditis (NVE) and prosthetic valve disease (PVE) and reported improved survival in patients with NVE [9]. This may possibly be due to increased disease severity in patients with PVE. Brenan et al. in their study at 605 centers within the Society of Thoracic Surgeons Adult Cardiac Surgery Database showed an elevated 12-year risk of reoperation and endocarditis amongst bio prosthesis patients [10]. In this study we analyzed the frequency of common etiological agents of IE using the highly sensitive standard automated system of blood culture methods (Bactec 9240). In addition, we assessed patient characteristics along with management outcomes amongst those with history of prior IVI labeled as high risk group (HRG) vs those without such risk factors termed as native valve group (NVG) and performed survival analysis for 90-day mortality. We hypothesized that HRG would be more likely to have severe disease outcomes, require further surgical interventions or readmissions, or die as opposed to conservative medical treatment.

## Methods

This was a prospective cohort study performed at the Clinical Laboratory (microbiology section) and cardiothoracic surgery of the Aga Khan University Hospital (AKUH), Karachi, Pakistan 2015–17. This is the biggest diagnostic laboratory set-up in Pakistan. We receive average of 1200–1500 blood culture samples per week from more than 200 outreach blood sample collection units in all major cities of Pakistan: hence the data represents country wide distribution.

We identified suspected IE patients from samples submitted at the clinical lab: all blood and relevant tissue samples (cardiac vegetation, cardiac valves, valvular abscess etc.) that were received with history suggestive of endocarditis were enrolled and verbal consent obtained for it. We followed blood / tissue culture to determine the microbiological and antimicrobial susceptibility profile of all recruited patients. The study was exempted from ethical approval by the research ethics committee under study number 3721-Pat-ERC-15 of the Aga Khan University hospital.

**Fig. 1:**
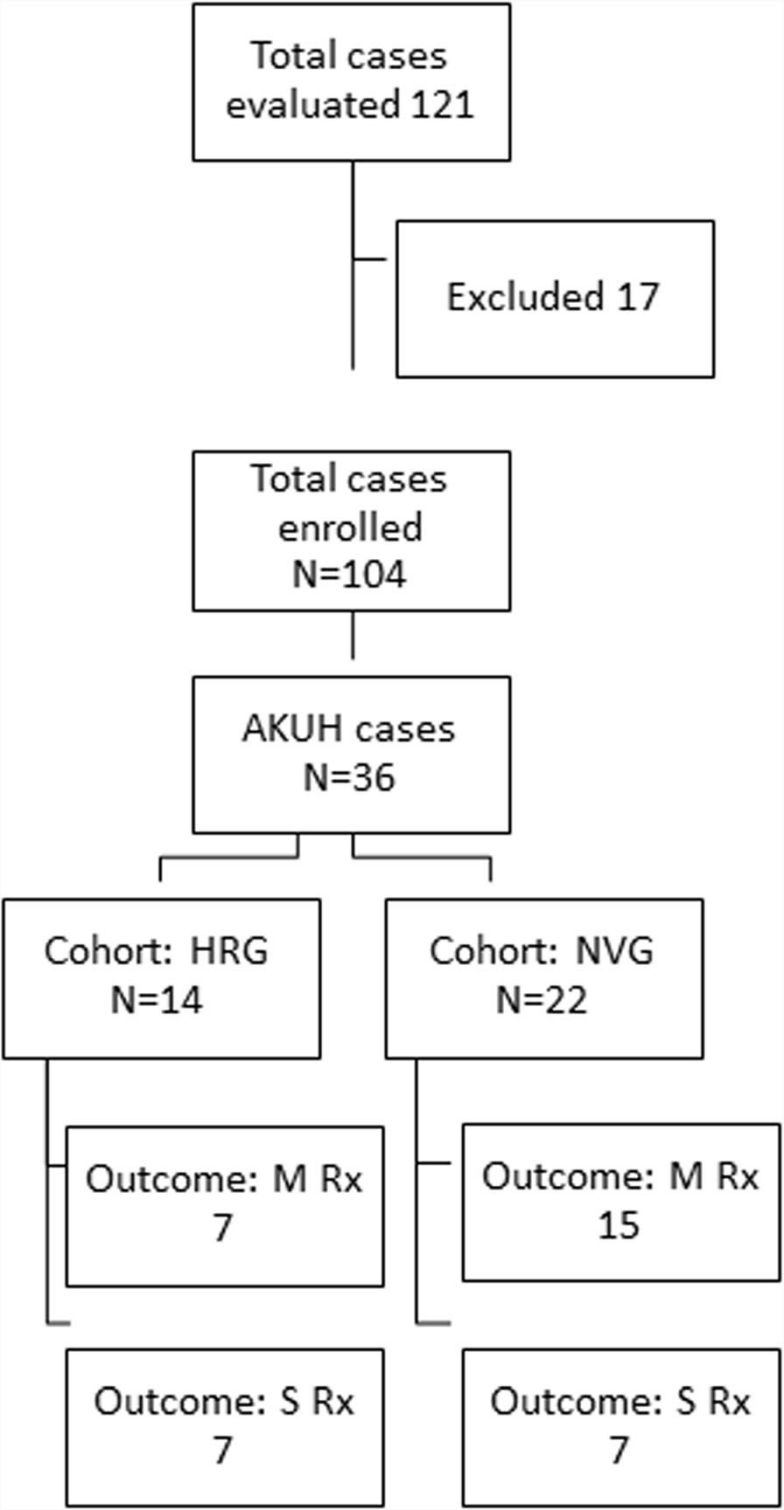
Study workflow for patient enrollment and group assignment. Description of study workflow for patient enrollment and assignment of groups for sub analysis (2015–2017). AKUH = Aga Khan University Hospital, HRG = High risk group, NVG = Native valve group, M Rx = Medical management, S Rx = Surgical management

In addition, we closely followed patients treated at AKUH; these patients were divided into two groups. A cohort of IE patients with prior history of prosthetic heart implants /prior cardiac intervention, valve repair/ replacements, Coronary artery bypass (CABG), angiography or angioplasty, correction of congenital cardiac vascular and valvular defects, pacemaker insertions within the last 12 years from time of enrollment in this study were classified as HRG and those without were labeled as NVG (see Fig. 1). To illustrate clinical characteristics and outcomes with respect to medical and surgical management, these two cohorts of patients were followed up till 90 days.

All suspected patients admitted under the service of internal medicine and cardio thoracic surgery fulfilling the modified Duke’s criteria (MoDC) for IE [2] were considered eligible and were enrolled. Eligible patients who could not be contacted for history were excluded. Any samples with damaged blood culture bottles or tissue in unsterile container were rejected and not included in the study. Details of patient’s clinical and microbiological profile were collected prospectively and entered in the predefined data collection form. Confidentiality was maintained by giving unique research identification number, and forms were kept under lock and key. Details included patient demographics, clinical presentation, duration of illness, use of prior antibiotics for the illness, predisposing cardiac condition, history of invasive cardiac procedures, microbiological and radiological findings along with echocardiography results; medical and surgical management details were recorded along with treatment outcome. Outcome in terms of disease severity was defined as any patient with IE undergoing surgical management, readmission or dying.

For microbiological profile, specimens were processed according to the standard techniques. Bactec 9240 automated system was used for blood culture as per Clinical & Laboratory Standards Institute (CLSI) protocol [11]. Tissue cultures were incubated for 21 days, antibiotic susceptibility testing was performed by disc diffusion method or Minimal inhibitory concentration (MIC) methods as recommended by CLSI and British Society for Antimicrobial Chemotherapy (BSAC) protocols, whichever was applicable. MIC was performed using Vitek2 system or E-strips as recommended. Microbiological outcome was determined as type / frequency of microorganisms isolated, antimicrobial susceptibility pattern of those isolates. All negative cases were reported “no growth” after incubation of 21 days.

### Statistical analysis

The data was analyzed using SPSS 21.0 software. In descriptive analysis mean and standard deviation of the continuous variables i.e. age, antibiotic MICs, duration of hospital stay etc. were calculated. Frequency and percentage of the categorical variables i.e. gender, etiological agent, antibiotic susceptibility pattern, clinical characteristics and surgical outcome were calculated. Risk of poor outcome was compared between HRG and NVG using chi-square test and difference was significant if *p*-value was ≤0.05. Kaplan Meier curves were generated to compare survival of HRG and NVG patients.

## Results

During the study period, 104 patients with clinical diagnosis of IE were prospectively enrolled (Fig. 1). Mean age of patients was 34.84 years with 72.1% being males. Adult representation was 84.6% and 15.4% were below 16 years. Using the MoDC 65.4% (*n* = 68) were identified as “definite cases of infective endocarditis” rest fell in the “possible case”. Cultures were sent on all 104 enrolled patients. Approximately 35% (*n* = 36) of patients were admitted at AKUH and were followed up for clinical outcomes.

Microbiological profile: Blood culture samples for laboratory diagnosis only, were received for 82.7% of the enrolled cases. Of these, 47.6% cases had 3 sets, while 14.6% had 2 sets of blood culture samples. For 10.6% cases both blood culture and cardiac tissues were received, while cardiac tissue or abscess pus aspirate samples without concomitant blood cultures were received in 6.7% of cases. Overall culture positivity rate was 71.2% (*n* = 74).

Among Gram positive organisms, *Streptococcus* species were the most common isolate (36.7%), predominantly *S. mitis*, followed by *Staphylococcus* species (23.1%). In *Streptococcus* species*,* 14.2% showed intermediate susceptibility to penicillin, (MIC50 = 0.12 μg/ml, MIC90 = 0.25 μg/ml); *S. oralis* being the species with the highest MIC range (0.06 μg/ml – 0.5 μg/ml). Half of the enterococcus isolates in the study were resistant to ampicillin, while all were sensitive to Vancomycin. *S. aureus* was isolated in 17.3% cases. Vancomycin MICs were performed only on Methicillin resistant strains of *S. aureus* (MIC range 0.5–1 μg/ml) and coagulase negative staphylococci (MIC 1 μg/ml). All MRSA strains were sensitive to Vancomycin. *Haemophilus actinomycetemcomitans, Klebsiella pneumoniae, Acinetobacter* species were isolated in 2.9% samples as primary pathogen and were found sensitive to third generation cephalosporin and carbapenem groups of antibiotics. Three patients had fungal endocarditis with *Candida albicans, Aspergillus niger, Fusarium* species, each. Table 1 shows details of frequency, type and antibiotic susceptibility of etiologic agents detected from blood and tissue cultures.

**Table 1:** Microbiological profile and antibiotic resistance pattern of isolates from patient with infective endocarditis received at the AKUH clinical laboratory (*n* = 104)

Microorganism	Total number cases *n* = 104(%)	Percent resistance of antibiotic for the species									
		CN	CI	ER	PE	VA	CH	OX	CP	AM	CR
Gram positive organism	**68 (65.4)**										
*Staphylococcus* species	**24 (23.1)**										
*•S. aureus*	18 (17.3)	27.0	22.2	50.0	100	00	00	77.	44.4	–	–
MSSA	4 (3.8)										
MRSA	14 (13.5)										
*•*CONS	6 (5.8)	16.6	16.6	33.3	100	00	00	83.3	60.0	–	–
*Streptococcus* species	**36 (34.7)**	NP	20.0	40.0	14.2	00	–	–	–	–	02.8
*•S. pneumoniae*	2 (1.9)										
*•S. mitis*	8 (7.7)										
*•S. oralis*	5 (4.8)										
*•S. sanguis*	3 (2.9)										
*•S. viridans*	3 (2.9)										
*•S. milleri*	1 (1.0)										
*•S. bovis*	3 (2.9)										
*•Streptococcus species*	5 (4.8)										
*•Granulicatella adiacens*	1 (1.0)										
*•Aerococcus viridans*	2 (1.9)										
*•Gemella haemolysan*	1 (1.0)										
*Enterococcus* species	**4 (3.8)**	25.0	–	50.0	50.0	00	25.0	**–**	**–**	50.0	**–**
*Corynebaterium* species	**4 (3.8)**	25	00	00	–	00	25.0	–	00		–
Gram negative organism	3 (2.9)	00	–	–	–	–	–	–	33.3	–	00
*Haemophilus actinomycetemcomitans, Klebsiella pneumoniae, Acinetobacter* species.											
Fungus	3 (2.9)	No resistance to azoles and Amphotericin									
*Candida albicans, Aspergillus niger, Fusarium* species											
Culture negative cases	30 (28.8)	–									

*CN* Gentamicin, *CI* Clindamycin, *ER* Erythromycin, *PE* Penicillin, *VA* Vancomycin, *CH* Chloramphenicol, *OX* Oxacillin, *CP* Ciprofloxacin *CR* Ceftriaxone, *MRSA* Methicillin resistant *Staphylococcus aureus*, *MSSA* Methicillin-Sensitive *Staphylococcus aureus*

### AKUH patient cohort analysis

Table 2 shows the frequency and association of patient demographics and clinical characteristics with HRG at the time of presentation. Table 3 presents the frequencies and risk of clinical outcomes with the HRG. Thirty six patients were included in the cohort analysis. The HRG included 14 patients whereas 22 were included in the NVG. Mean age of patients in HRG was 45 years and 38 years in NVG. Male predominance was seen in both groups. Eighty five percent cases in HRG were diagnosed as definite IE, 41.6% presenting with acute symptoms. In the NVG, 72.2% were diagnosed as definite IE with acute symptoms in 54.4% patients. Culture positivity rate was 71.4% in HRG and 90.9% in NVG. RHD was seen in 18.1% cases of NVG only. In the HRG, prosthetic cardiac valve and implants (stents, pacemakers) were seen in 78.5% cases. A high proportion of HRG patients had congenital heart disease (42.8%), who underwent invasive intervention in the past. Most patients presented with fever. Breathlessness was seen in 35.7% HRG and 54.5% NVG. Edema was present in 18% NVG patients. Signs of sepsis were more frequent in NVG (27.2% vs. 7.1% in HRG). Multi organ dysfunction including acute renal injury (AKI), splenomegaly, hepatomegaly, lymphadenopathy, pulmonary symptoms were seen in 31.81% of NVG vs 28.58% in HRG. Murmur was present in 36.6% NVG patients. Presence of vegetation was seen in almost all patients in HRG (92.8%) as shown in Table 2. Although too small a number to be significant, mitral valve prolapse was seen only in the NVG (13.6%) and pulmonary regurgitation only in HRG (14.2%).

**Table 2:** Frequency and association of patient demographics, clinical characteristics and underlying disease severity with HRG at the time of presentation of IE patients admitted at AKUH 2015–2017 (n = 36)

PATIENT CHARACTERISTICS	HRG *N* = 14	NVG *N* = 22	*p*-value	Odds Ratio (Confidence interval)
Mean Age	45	38	–	–
Gender: Male	8	15	0.501	0.838 (0.490–1.432)
MoDC				
*•*Definite	12	16	0.361	1.179 (0.844–1.645)
*•*Probable	2	6	0.361	0.524 (0.122–2.240)
Acute clinical presentation	5	12	0.270	0.655 (0.294–1.457)
Sub-acute presentation	9	10	0.270	1.414 (0.775–2.581)
Positive culture	10	20	0.126	0.786 (0.550–1.122)
Pure culture growth	14	18	0.091	1.222 (1.004–1.488)
Rheumatic heart disease	0	4	0.090	
Prosthetic heart valve / cardiac implants	11	0	–	–
Congenital heart disease	6	5	0.201	1.886 (0.708–5.022)
Severity at presentation				
Signs of thromboembolism^a^	2	7	0.236	0.449(0.108–1.860)
Sepsis	1	6	0.137	0.262 (0.951–1.714)
Multi organ dysfunction	4	7	0.837	0.898 (0.321–2.514)
Rash /splinter hemorrhage	0	4	0.091	–
*•*Previous history of infective endocarditis	1	0	0.204	–
*•*Murmur	2	8	0.149	0.393 (0.920–1.972)
*•*Raised infectious markers	1	2	0.837	0.786 (0.078–7.876)
Radiologic evidence on Echocardiography				
*•*Vegetation	13	14	***0.048***	*1.45 (1.03–2.066)*
*•*Mitral valve prolapse	0	3	0.149	–
*•*Peri-annular abscess	2	3	0.956	1.048 (0.199–5.504)
*•*Valve perforation / dehiscence	1	1	0.740	1.571 (0.107–23.140)
*•*Portal hypertension	8	11	0.826	–
*•*Aortic regurgitation	6	10	0.878	0.943 (0.442–2.013)
*•*Mitral regurgitation	4	7	0.837	0.898 (0.321–2.514)
*•*Pulmonary regurgitation	2	0	0.068^a^	–

*HRG* High risk group, *NVG* Native valve group, *MoDC* Modified Dukes Criteria

^a^= gangrene, unilateral weakness, slurring of speech, loss of consciousness

**Table 3:** Clinical outcome characteristics of IE patients admitted at AKUH 2015–2017 (*n* = 36)

CLINICAL OUTCOME	HRG *N* = 14	NVG *N* = 22	*p*-value	RISK
Disease severity	12	11	***0.030***	***1.714 (1.072–2.741)***
*•*Surgical management	7	15	0.275	1.571 (0.702–3516)
*•*Readmission	5	2	0.143	–
*•*Death	2	5	0.533	0.629 (.0141–2.808)
Presence of embolism / infarct	2	10	***0.05***	***0.314 (0.080–1.227)***
*•*Infarct	0	4	0.091	–
*•*Embolism	2	6	0.361	0.524 (0.122–2.240)
○Brain	0	6	***0.032***	–
○Lung	1	4	0.350	0.393 (0.049–3.165)
○limbs	1	0	0.204	–
Complication during hospital stay	5	6	0.592	1.310 (0.492–3.488)
Sepsis	1	1	0.740	1.571 (0.107–23.140)
*•*Coagulation / Hematological issue	2	2	0.629	1.571 (0.249–9.913)
*•*Multi-organ dysfunction^a^	2	1	0.303	3.143 (0.314–31.506)
*•*Re- exploration	1	2	0.837	0.786 (0.078–7.876)
*•*Cardiac conduction defects	1	1	0.740	1.571 (0.107–23.140)
Indication for Surgical Intervention				
*•*Sepsis	2	2	0.629	1.571 (0.249–9.913)
*•*Embolism	0	2	0.246	–
*•*Large vegetation	9	5	***0.013***	***2.829 (1.192–6.710)***
*•*Cardiac failure	0	4	0.091	–
Valve replacement	**6**	**8**	***0.048***	
Cause of Death	–	–	0.852	–
*•*Cardiac arrest	1	2	0.852	–
*•*Cerebral bleed	0	1	0.143	–
*•*Sepsis	1	2	0.533	0.629 (.0141–2.808)

*HRG* High risk group, *NVG* Native valve group

^a^= acute renal injury (AKI), splenomegaly, hepatomegaly, lymphadenopathy, pulmonary symptoms

**Fig. 2:**
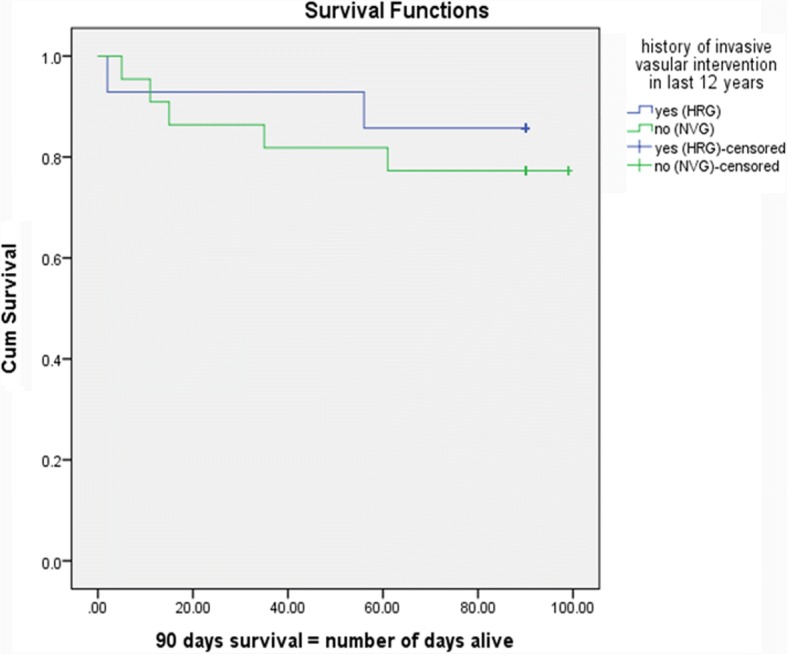
Kaplan Meier survival curves for high risk and native valve group. The Kaplan Meier curves show difference between the survival outcomes of High risk group (HRG) and native valve group (NVG) which is statistically insignificant (*p*-value = 0.629)

Regarding clinical outcomes, the risk of disease severity was higher amongst the HRG than NVG (Table 3), with 85.7% HRG patients having poorer outcome compared to 50% of NVG. However, more deaths were seen in the native valve group (22.72%), despite not achieving statistical significance probably due to small number of events. The Kaplan Meier curve is shown in Fig. 2. The risk of embolism or infarct was higher with NVG (45.5%); the involvement of central nervous system seen exclusively in this group (27.7%). Thirty six percent cases of the HRG had complications during hospital stay (NVG = 27.2%). Large vegetation was the most common indication for surgery in both groups (HRG = 64.2%, NVG = 22.7%), but significantly associated with HRG.

Additional file 1: Table S4 shows clinical presentations and outcomes specific to the organisms isolated amongst Infective Endocarditis patients admitted at AKUH 2015–2017. *Streptococcus* spp. was equally prevalent in both groups (22.7% in NVG vs 21.4% in HRG). In the NVG, cases presented with murmur, thromboembolic (TE) event, rash, cardiac tamponade and abscess (*S. oralis)* on echocardiograph at presentation. All these cases were treated medically and had Penicillin MIC range of 0.03–0.5 μg/ml. There was one death; the patient’s isolate had intermediate susceptibility to Penicillin with MIC of 0.12 μg/ml. In the HRG, 3 cases of *Streptococcus* species were seen; Penicillin MIC range was 0.015–0.012 μg/ml. Only one presented with an abscess and the infecting organism in this case was *S. sanguis*. *S.aureus* was seen more in the NVG compared to HRG. MRSA endocarditis cases presented with TE event, AKI, raised infectious markers, murmur, splenomegaly, rash, sepsis and splinter hemorrhages and only two were treated surgically. Amongst the 3 mortalities, 2 received medical treatment only while one died at day 11 of hospital admission 24 h post valvuloplasty. The patient presented with signs of acute IE with MRSA (vancomycin MIC 1 μg/ml) and developed multiorgan dysfunction and pulmonary embolism. This patient had multiple vegetations (11x11mm, 9x7mm) on Tricuspid valve and underwent valve replacement due to heart failure and sepsis. Infections with coagulase negative staphylococcal species was seen exclusively in the HRG (*n* = 3), presenting with TE event, AKI, raised infectious markers, splenomegaly and abscess on echocardiograph. Two of these patients were treated surgically and 1 medically, who later expired.

## Discussion

In the 2-year study period, we enrolled 104 patients of IE, of which 65.4% were definite cases based on MoDC. Of enrolled cases 28.8% were culture negative as compared with 50% previously reported in studies from Pakistan [7, 8]. The most plausible explanation for this difference is the sensitivity of blood and tissue culture methods used in this study. Most etiological agents of IE are fastidious and require highly nutrient culture medium and controlled incubation conditions. BACTEC 9240 system of blood culture is known to have better culture yield [12] as compared to conventional blood culture method used in previous studies from Pakistan. However when compared with data from western studies reporting blood culture yield of 90% [13] our culture yield of 74.2% is much lower. The most compelling reason for this low yield are the pre-analytical factors, such as use of antibiotics prior to blood culture collection. This was noticeable in 71% of all enrolled cases in current study. Another important factor is the adequate volume of blood cultured. Since density of microorganisms in blood is often very low, adequate volume and multiple sampling is considered to be an important parameter for improved blood culture yield. Guidelines from most professional societies, such as American Society for Microbiology (ASM) and Infectious Disease Society of America (IDSA), recommend that adult patient with suspected IE must be investigated by drawing at least 3 blood culture sets with appropriate volume [1]. In our study population only 47.3% of total patients recruited had 3 sets of blood culture. Inaccessibility to health facilities, increasing diagnostic cost and lack of awareness are factors that often contribute to poor compliance to these essential pre-analytical components of blood culture analysis in Pakistan.

Contrary to previous reports of predominantly younger age group [3, 7] we found 15.4% of enrolled patients being below age of 16 years. Tariq et al. in his study conducted in 2004 reported mean age of 24 years [3], same group in 2015 has reported a shift in mean age 42 years [4], close to the mean age of patients in this study (34.5 years). This progressive increase perhaps reflects the shift in the underlying risk factors. During the last two decades reports from Pakistan are showing a gradual shift from communicable to non-communicable diseases (NCDs) such as cardiovascular diseases (including stroke and heart disease), diabetes, mental health disorders, cancers, and chronic airway diseases [14]. Management of most of these diseases often requires advanced medical care such as use of cardiac implants, invasive vascular interventions etc., predisposing patients to risk factors of endocarditis similar to those of the modern world –however more data is required to verify this change.

We found *Streptococcus* group of bacteria to be the most frequently isolated organisms from blood and tissue cultures in both groups. These findings are similar to those published by other groups nationally and from neighboring countries like China, India [3, 5, 7, 15–17] as well as internationally [2, 18]. Although *Streptococci* seem to predominate in developing regions, most western and developed parts of the world report *S. aureus* as the predominant causative agent of IE [19, 20]. *S. mitis* was the most common species isolated similar to a recent survey involving 118 hospitals in Japan reporting *S.viridians* as the predominant species in NVE [21]. Penicillin remains drug of choice against this group of bacteria however we found 14.2% of our isolates to be intermediately resistant with MIC as high as 1 μg/ml. No particular species of *S.viridans* predominantly showed higher MIC. The numbers of isolates in our study were too few to establish such association. A study in USA reported high-level penicillin resistance among 13.4% and intermediate resistance in 42.9% strains of *S. viridans* [22].To the best of our knowledge no prospective study in Pakistan relates pathogens and their respective MICs with disease severity in IE, a finding unique to our study.

Of the 18 *S. aureus* isolated, 77.7% were MRSA and were more frequent in the NVG. MRSA infections showed increase disease severity at time of presentation such as TE events, AKI, raised infectious markers, murmur splenomegaly, rash, sepsis and splinter hemorrhages. It was the commonest pathogen found amongst expired patients in both cohorts. A study in Turkey reported 18% of IE deaths due to endocarditis, a number higher than any other pathogen isolated [23]. This could be due to the high prevalence of MRSA in the region. Asia has higher prevalence of both healthcare-associated methicillin-resistant *Staphylococcus aureus* (HA-MRSA) and community-acquired methicillin-resistant *S. aureus* (CA-MRSA) [24].

Human brucellosis is common in Pakistan in patients with risk factors such as animal exposure, use of unpasteurized milk etc. Blood cultures positive for patients suffering from Brucella infections are often reported from this lab, however none of the cultures in this study yielded Brucella sp., as a cause of endocarditis. This could be because of selection bias of our patients as most of the samples recruited in the study were from patients under cardiac care. In addition, we had limitation of non-availability of methods such as PCR and serological analysis.

Embolism is not uncommon and risk is seen in 22–57% cases of IE: the risk progressively increased with the size of vegetation [25] . Amongst our patients 12 presented with evidence of embolism or infarct. Of these 6 were cerebral, 5 pulmonary and 1 with peripheral involvement. A prospective multicenter European study presented 34.1% cases with true embolic events [20]. Most were involving the CNS similar to our findings. Statistically significant difference was seen between the 2 cohorts with more embolic events in the NVG. A retrospective study in France presented embolism in 62% of PVE vs. 35% of NVE patients [25]. Although this contradicts our findings, this could be due to delay in diagnosis as patients with prior invasive intervention or cardiac anomalies tend to access tertiary care earlier. Embolism is directly related to the size of vegetations, therefore late presentations are likely to have larger vegetations.

The mortality rate in our study was 19.4% and we saw more deaths and more severe case presentations in NVG as opposed to HRG (Table 3). This is most likely due to inaccessibility to health facilities, increasing diagnostic cost leading to delay in diagnosis. HRG was associated with severe disease outcomes, a statistically significant finding despite a small sample size. These were mainly due to higher rates of surgical intervention and readmission due to complications. In a retrospective review of surgical patients with NVE and PVE, Manne et al. described a severe clinical course amongst those with PVE, with 23% 1 year mortality rate, higher risk of post-operative complication like cerebrovascular accident (3.3%), renal failure (11%), reoperation (9.4%) [9].

Our study had some limitations which included a small sample size and convenient sampling. Ideally, an additional control group comprising of uninfected surgical cases should have been included to remove confounding by surgical complications from those of complicated IE. The sample size was small to establish statistically significant findings. However, a larger sample size was not possible due to the low prevalence and short duration of this prospective study.

Secondly, we could not ascertain culture negative cases as we did not perform additional testing for organisms like *Bartonella* spp., *Coxiella burnetti* and *Tropheryma whipplei* due to lack of technical facilities and availability of reagents and positive control strains.. However for *Mycobacterium tuberculosis* culture was performed in selected patients; cardiac tissue and pus aspirates when requested by the clinicians, and in this study all MTb cultures remained negative. Being a single center study, unrecognized confounding factors and selection bias may have affected results. This study is strengthened by its prospective nature as well as description of clinical details and disease outcomes relating to pathogens in IE patients.

## Conclusion

In conclusion our findings support the hypothesis that HRG can encounter a more complicated clinical course requiring further surgical interventions, readmissions or death as opposed to native valve endocarditis patients therefore we recommend close follow up of high risk population.

## Additional file

Additional file 1: **Table S4.** Risk profile and outcomes specific to the organisms isolated amongst. Infective Endocarditis patients admitted at AKUH 2015–2017 (*n* = 36). (DOCX 14 kb)

## Abbreviations

AKI: Acute renal injury
AKUH: Aga Khan University Hospital
ASM: American Society for Microbiology
BSAC: British Society for Antimicrobial Chemotherapy
CABG: Coronary artery bypass
CA-MRSA: Community-acquired methicillin-resistant *S. aureus*
CH: Chloramphenicol
CI: Clindamycin
CLSI: Clinical & Laboratory Standards Institute
CN: Culture negative
CN: Gentamicin
CP: Ciprofloxacin
CR: Ceftriaxone
ER: Erythromycin
GNO: Gram negative organism
GPB: Gram positive bacilli
HA-MRSA: Healthcare-associated methicillin-resistant *Staphylococcus aureus*
HRG: High risk group
IDSA: Infectious Disease Society of America
IE: Infective Endocarditis
IVI: Invasive vascular interventions
MIC: Minimal inhibitory concentration
MoDC: Modified Duke’s criteria
MRSA: Methicillin-resistant *Staphylococcus aureus*
MSSA: Methicillin-Sensitive *Staphylococcus aureus*
NCDs: Non-communicable diseases
NVE: Native valve endocarditis
NVG: Native valve group
OX: Oxacillin
PE: Penicillin
PVE: Prosthetic valve disease
RHD: Rheumatic heart disease
TE: Thromboembolic
VA: Vancomycin
