# Supplementary material for: Microbiological and clinical profile of infective endocarditis patients: an observational study experience from tertiary care center Karachi Pakistan
Source: J Cardiothorac Surg. 2018 Sep 15;13:94. doi: 10.1186/s13019-018-0781-y (PMC6139130; doi:10.1186/s13019-018-0781-y)
Supplement: Supplementary file 1 — Table S4. Risk profile and outcomes specific to the organisms isolated amongst. Infective Endocarditis patients admitted at AKUH 2015–2017 (n = 36). (DOCX 14 kb) [file 13019_2018_781_MOESM1_ESM.docx]

Table S4 Risk profile and outcomes specific to the organisms isolated amongst Infective Endocarditis patients admitted at AKUH 2015- 2017 (n=36)

| **NATIVE VALVE GROUP (n=22)** | | | | | |
| --- | --- | --- | --- | --- | --- |
| **ORGANISM** | **n** | **RISK PROFILE AT TIME OF ADMISSION** | **S Rx** | **DEATH** | **CAUSE OF DEATH** |
| *Streptococcus spp.* | 5 | Murmur, Thromboembolic event, rash, cardiac tamponade, Abscess on echocardiograph (*S. oralis*) | 0 | 1 | cerebral bleed |
| *Enterococcus spp.* | 1 | Thromboembolic event | 0 | 0 | - |
| MRSA | 7 | Thromboembolic event, AKI, raised infectious markers, murmur Splenomegaly, rash, sepsis, splinter hemorrhages | 2 | 3 | cardiac arrest  sepsis |
| MSSA | 2 | Thromboembolic event, sepsis, Abscess on echocardiograph | 0 | 0 |  |
| GPB group | 3 | Splenomegaly, murmur, Abscess on echocardiograph (*Propionibacterium acnes*) | 2 | 0 |  |
| GNR group | 1 | Thromboembolic event, raised infectious marker, murmur | - | 0 |  |
| Fungus | 0 | - | - | 0 |  |
| CN | 3 | Sepsis, AKI, murmur, loss of consciousness | 11 | 1 | cardiac arrest |
| **HIGH RISK GROUP (n=14)** | | | | | |
| *Streptococcus spp.* | 3 | Abscess on echocardiograph (*S. sanguis*) | 1 | 0 |  |
| *Enterococcus spp.* | 1 | - |  |  |  |
| MRSA | 2 | Rash, Thromboembolic event, hepatomegaly | 1 | 0 |  |
| MSSA | 0 | - | - | - | - |
| CoNS | 3 | Thromboembolic event, AKI, raised infectious markers, Abscess on echocardiograph, splenomegaly | 2 | 1 | cardiac arrest |
| GPB | 1 | - | 1 | 0 | - |
| GNR | 0 | - | - | - | - |
| Fungus (*Fusarium spp.)* | 1 | Previous history infective endocarditis , Uncontrolled sepsis |  | 1 | Sepsis |
| CN | 3 | Lymphadenopathy | 2 | 0 | - |

AKI= Acute kidney injury, CN= Culture negative, CoNS= Coagulase Negative Staphylococci, GNR= Gram negative rods, GPB= Gram positive bacilli, MRSA*=* M**ethicillin Resistant *Staphylococcus aureus*, MSSA=** Methicillin Sensitive *Staphylococcus aureus,* S Rx= Surgical management
